# Comparison of peptide–major histocompatibility complex tetramers and dextramers for the identification of antigen-specific T cells

**DOI:** 10.1111/cei.12339

**Published:** 2014-06-09

**Authors:** G Dolton, A Lissina, A Skowera, K Ladell, K Tungatt, E Jones, D Kronenberg-Versteeg, H Akpovwa, J M Pentier, C J Holland, A J Godkin, D K Cole, M A Neller, J J Miles, D A Price, M Peakman, A K Sewell

**Affiliations:** * Institute of Infection and Immunity, Cardiff University School of MedicineCardiff, UK; † Department of Immunobiology, School of Medicine, King's College LondonLondon, UK; ‡ QIMR Berghofer Medical Research InstituteBrisbane, QLD, Australia; § School of Medicine, The University of QueenslandBrisbane, QLD, Australia; ¶ Human Immunology Section, Vaccine Research Centre, National Institute of Allergy and Infectious Diseases, National Institute of HealthBethesda, MD, USA

**Keywords:** autoimmunity, diabetes, T cell receptors, T cells, tumour immunology

## Abstract

Fluorochrome-conjugated peptide–major histocompatibility complex (pMHC) multimers are widely used for flow cytometric visualization of antigen-specific T cells. The most common multimers, streptavidin–biotin-based ‘tetramers’, can be manufactured readily in the laboratory. Unfortunately, there are large differences between the threshold of T cell receptor (TCR) affinity required to capture pMHC tetramers from solution and that which is required for T cell activation. This disparity means that tetramers sometimes fail to stain antigen-specific T cells within a sample, an issue that is particularly problematic when staining tumour-specific, autoimmune or MHC class II-restricted T cells, which often display TCRs of low affinity for pMHC. Here, we compared optimized staining with tetramers and dextramers (dextran-based multimers), with the latter carrying greater numbers of both pMHC and fluorochrome per molecule. Most notably, we find that: (i) dextramers stain more brightly than tetramers; (ii) dextramers outperform tetramers when TCR–pMHC affinity is low; (iii) dextramers outperform tetramers with pMHC class II reagents where there is an absence of co-receptor stabilization; and (iv) dextramer sensitivity is enhanced further by specific protein kinase inhibition. Dextramers are compatible with current state-of-the-art flow cytometry platforms and will probably find particular utility in the fields of autoimmunity and cancer immunology.

## Introduction

The use of fluorochrome-conjugated peptide–major histocompatibility complex (pMHC) multimers in conjunction with flow cytometry has become commonplace methodology for studying antigen-specific T cell populations 1,2. pMHC multimers offer several advantages over other technologies for the identification of antigen-specific T cells, as they can detect T cells regardless of their effector function and without the requirement for cellular activation. This feature enables phenotyping of T cells directly *ex vivo* by using a spectrum of fluorochrome-conjugated antibodies specific for other T cell markers. More recent advances have expanded possibilities by using heavy atom-conjugated pMHC multimers and antibodies in combination with mass spectrometry 3. The success of pMHC multimers is demonstrated by their use in thousands of published studies and the commercialization of several different pMHC multimerization platforms 1. The original such platform, an avidin–biotin-based tetramer 4, is still the most common format in use, as it can be readily manufactured in laboratories equipped with basic protein expression and purification facilities. However, several other multimerization strategies are now available to investigators, including pMHC pentamers and octamers 1. Dextran-based pMHC multimers (dextramers) are a more recent addition to this toolbox 5.

We have determined previously that the binding affinity threshold for pMHC class I (pMHC-I) tetramers is significantly higher than that required for T cell activation 6. As a result, pMHC-I tetramers can often fail to stain antigen-specific T cells where the interaction between pMHC and T cell receptor (TCR) is weaker than K_D_ = 80 μM. Such weak TCR–pMHC affinities are not usually characteristic of CD8^+^ T cells specific for foreign, pathogen-derived antigens, and pMHC-I tetramers have excelled when used to characterize virus-specific cytotoxic T lymphocyte (CTL) populations. In contrast, the use of pMHC-I tetramers can be more problematic when the reagents are used to identify T cells specific for self-derived peptides (anti-tumour and autoimmune T cells). Self-reactive T cells are known to generally bear weaker binding TCRs 7. This is thought to be the result of thymic editing that culls T cells bearing higher-affinity self-reactive TCRs 7. Thus, at present, pMHC-I tetramers cannot be used to detect all antigen-specific CD8^+^ T cells 1,6. There are further issues with the use of pMHC class II (pMHC-II) tetramers for the detection of T helper cells 1,8. First, antigen-specific CD4^+^ T cell populations tend to be considerably smaller in number than the larger anti-viral CD8^+^ T cell populations. Secondly, MHC-II-restricted TCRs bind with approximately fivefold lower affinity than MHC-I-restricted TCRs 7. Thirdly, while the CD8 molecule stabilizes TCR–pMHC-I interactions through effects on both the TCR on-rate and off-rate, the CD4 co-receptor does not contribute to TCR–pMHC-II binding 1,9–15. The lower affinity of TCR–pMHC-II interactions combines with the lack of CD4 ‘co-receptor help’ to ensure that the average CD4^+^ T cell is almost 50-fold more difficult to stain with pMHC tetramers than the average CD8^+^ T cell 1. Thus, there is a currently a pressing need to extend pMHC multimer technology to a point where it can be used to stain *all* antigen-specific T cells in *all* biological systems. This requirement is greatest for MHC-II-restricted T cells and most T cells that target self-derived peptides 16.

Several improvements of pMHC multimer technology can be used to extend the range of TCR–pMHC interactions that are amenable to detection. The use of higher concentrations of pMHC multimer can, to some extent, aid staining where TCR–pMHC interactions are weak 1. However, such use is not economically attractive and can result in concomitant increases in background staining. It is also established that anti-co-receptor antibodies can regulate pMHC multimer binding 1,11,13,14,17. In general, researchers now stain with pMHC multimer prior to co-receptor staining. It has also been established that some murine and human anti-CD8 antibodies act to enhance the capture of pMHC-I multimer from solution, so the use of anti-co-receptor antibodies that enhance pMHC multimer staining is recommended 18. It is possible to further increase the range of TCR–pMHC interactions that are amenable to pMHC-I tetramer staining by using CD8-enhanced tetramers 13. However, enhancing the universal pMHC-I–CD8 interaction could potentially result in an artificial increase in the degeneracy of TCR binding 12. The more recent procedure of including a non-toxic, reversible protein kinase inhibitor (PKI) during pMHC multimer staining can also increase the range of TCR–pMHC interactions that can be detected with pMHC multimers without altering the TCR–pMHC or pMHC–CD8 interactions 19. This simple technology can result in >50-fold improvements in the staining of T cells bearing very weak binding TCRs, and is universally applicable to pMHC multimer staining of CD8^+^ and CD4^+^ human, other primate or murine T cells 1,19.

To address these evident technology gaps, we have evaluated the use of pMHC-I and pMHC-II tetramers and dextramers across a range of systems. Specifically, we aimed to examine whether pMHC dextramers could be used to stain T cells with a low TCR–pMHC affinity and whether this novel technology was preferable to classical pMHC tetramers for the detection of tumour-specific, autoimmune and pMHC-II-restricted T cell populations that tend to display weaker affinity TCRs and are undetectable by conventional means.

## Materials and methods

### Cells and cell lines

The following human leucocyte antigen (HLA)-A*0201 (HLA-A2)-restricted CD8^+^ T cell clones were used: (i) ILA1, which recognizes the ILAKFLHWL epitope from human telomerase reverse transcriptase (hTERT: residues 540–548) 20, in addition to four altered peptide ligands (APL) to which the ILA1 TCR has varying affinities 21; (ii) NLV2F3, which recognizes the NLVPMVATV epitope from cytomegalovirus (CMV) pp65 (residues 495–503); and (iii) 3F2 and 1E6, which recognize the ALWGPDPAAA epitope from preproinsulin (PPI: residues 15–24) and originate from a patient with type 1 diabetes (T1D) 22,23. The HLA-A*2402 (HLA-A24)-restricted clone, 4C6, was also generated from a patient with T1D and recognizes the LWMRLLPLL epitope from PPI (residues 3–11) 24. Additionally, the HLA-DRB1*0101 (HLA-DR1)-restricted CD4^+^ clone, Flu2C5, was used, which recognizes the PKYVKQNTLKLAT epitope from influenza A haemagglutinin (HA: residues 307–319) 25. T cell clones were routinely expanded by restimulation with allogeneic peripheral blood mononuclear cells (PBMCs) and phytohaemagglutinin (PHA), as described previously 26, then cultured for at least 2–3 weeks before being used for experimentation. Fresh heparinized blood was obtained from volunteers or via the Welsh Blood Service, with appropriate ethical approval. PBMCs were isolated by standard density centrifugation, by layering blood over an equal volume of lymphoprep (Axis Shields, Dundee, UK). PBMCs were either used immediately or from cryopreserved samples, with the latter being treated with 10–50 μg/ml of DNase I (Roche, Burgess Hill, UK) for 30 min at 37°C after thawing. Spiked samples were created by mixing clonal T cells (10^4^) with PBMCs (10^6^). The spiked PBMCs were minimally HLA-matched for the restricting HLA of the spiking clone.

### Tetramer and dextramer assembly

Soluble biotinylated pMHC-I and pMHC-II monomers were produced as described previously 15, with the same monomeric pMHC protein being used to make paired tetramers and dextramers for direct comparison. Tetramers were assembled by the stepwise addition of R-phycoerythrin (PE), allophycocyanin (APC) or Alexa Fluor 488 (Alexa488)-conjugated streptavidin (all from Life Technologies, Paisley, UK) at a molar pMHC : streptavidin ratio of 4:1. Dextran–streptavidin–fluorochrome conjugates were provided by Immudex Limited (Copenhagen, Denmark) and consist of a dextran polymer scaffold with covalently linked fluorochrome and streptavidin. Individual dextramers conjugated to PE, APC and fluorescein isothiocyanate (FITC) averaged 3, 7 and 33 fluorochrome molecules and typically, 6, 3 and 13 streptavidins with the typical number of streptavidins per dextramer exhibiting the expected Gaussian distribution. The amount of input streptavidin was determined by absorbance at 280 nm. After reaction with the dextran backbone and purification, the amount of streptavidin in the dextran-conjugate and ‘supernatant’ (free streptavidin) was measured by absorbance at 280 nm and used to calculate the number of strepavidin molecules per dextran molecule for each batch of dextramer ‘backbone’. Dextramers (dextramer backbone and pMHC) were assembled by the addition of monomeric pMHC at a molar ratio of 3:1 (APC and PE) or 1:1 (FITC), with respect to the streptavidin component of the dextramer backbone, for 30 min at room temperature. Once assembled, tetramer and dextramer were standardized to a working concentration of 0·1 mg/ml in phosphate-buffered saline (PBS) or dextramer buffer (0·05 M Tris-HCL, 15 mM NaN_3_, 1% bovine serum albumin, pH 7·2) filtered at 0·2 μm, respectively. Protease inhibitors (Set 1; Merck, London, UK) were then added and the reagents stored at 4°C in the dark.

### Cell staining and flow cytometry

**Fig. 1:**
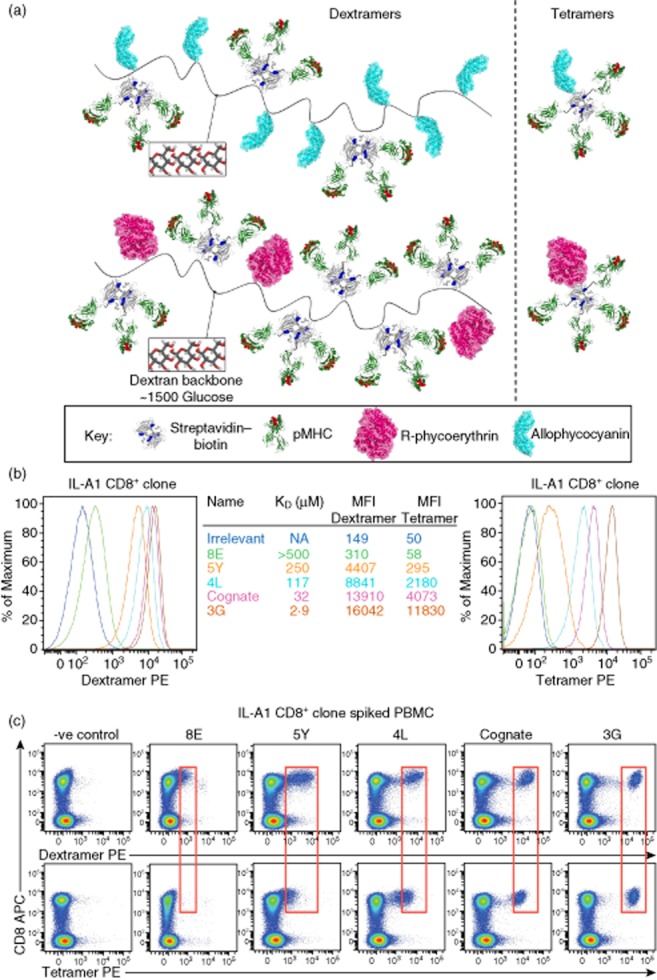
Peptide–major histocompatibility complex (pMHC) dextramers bind CD8^+^ T cells substantially better than tetramers when T cell receptor (TCR)–pMHC affinity is low. (a) A schematic showing how pMHC dextramers differ from pMHC tetramers. Dextramers have a dextran backbone and carry more fluorochrome and pMHC per molecule. The fluorochrome/pMHC ratio differs between allophycocyanin (APC) and R-phycoerythrin (PE) dextramers. Figure 1(a) is available as a separate high-resolution file (Supporting Information Fig. S3) in the online supplement that accompanies this article. (b) The ILA1 CD8^+^ T cell clone was stained with PE-conjugated tetramer (right) or dextramer (left) reagents constructed with human leucocyte antigen (HLA)-A2 monomers bearing the wild-type human telomerase reverse transcriptase (hTERT)_540–548_ peptide (ILAKFLHWL), variants thereof with defined TCR–pMHC-I affinities as indicated, or the HIV-1 Pol_476–484_ peptide (ILKEPVHGV) as an irrelevant control. (c) Human peripheral blood mononuclear cells (PBMCs) (10^6^) were spiked with clonal ILA1 cells (10^4^) and stained with the indicated tetramers (bottom) or dextramers (top), the viability dye Aqua and monoclonal antibodies against the surface markers CD3, CD4, CD8, CD14 and CD19. Gates were set serially on lymphocytes, single cells and live CD3^+^CD14^−^CD19^−^ cells prior to display as CD8 *versus* tetramer/dextramer. In each column, the red box is positioned on the antigen-specific CD8^+^ T cell population that stains most brightly to aid visual comparison.

All tetramers, dextramers and monoclonal antibodies conjugated to either quantum-dot (QD) 705 or APC-H7 were microcentrifuged for 1 min before use to remove aggregates. A total of 5–10 × 10^4^ of a T cell clone or 2–4 × 10^6^ of PBMCs (spiked or unspiked) were stained with a desired amount of tetramer (range between 1·25–40 μg/ml with respect to the pMHC component) or dextramer (range between 0·74–24 μg/ml with respect to the pMHC component) in 50 μl of staining buffer [PBS supplemented with 2% fetal bovine serum (FBS) or 1% human serum and 25 mM HEPES buffer]. Routine stains were performed using 0·5 μg of tetramer (10 μg/ml) or 0·3 μg of dextramer (6 μg/ml) per sample for 30 min on ice to ensure optimal discrimination (Fig. 1 and Supporting Information Fig. S1). All cell samples were subsequently stained with a LIVE/DEAD amine-reactive dye (Violet or Aqua; Life Technologies) for 5 min at room temperature. In some instances, clonal T cells were stained further with anti-CD4 (αCD4) (VIT4; Miltenyi Biotec, Bergisch Gladbach, Germany; SK3, BD Biosciences) or αCD8 (BW135/80, Miltenyi Biotec; SK1, BD Biosciences, Oxford, UK) antibodies for 20 min on ice. PBMCs (spiked or unspiked) were stained further with αCD14 (M5E2; BioLegend, London, UK), αCD19 (H1B19, BioLegend), αCD3 (BW264/56, Miltenyi Biotec; SK3, BD Biosciences) and/or αCD4/αCD8 antibodies for 20 min on ice. For immunophenotyping experiments, PBMCs prelabelled with tetramer or dextramer were stained with LIVE/DEAD Violet (ViViD; Life Technologies) as above and a panel of directly conjugated monoclonal antibodies as follows: (i) αCD14 Pacific Blue (TüK4), αCD19 Pacific Blue (SJ25-C1), αCD8 QD705 (3B5) and αCD69 APC (CH/4) (Life Technologies); (ii) αCD3 APC-H7 (SK7), αCD57 FITC (NK-1) and αCCR7 PE-Cy7 (3D12) (BD Biosciences); and (iii) αCD27 PE-Cy5 (1A4CD27) and αCD45RO ECD (UCHL1) (Beckman Coulter, High Wycombe, UK). Typically, PBMC samples were gated serially on single, amine/CD14/CD19 (dump)-negative, CD3^+^ lymphocytes and displayed in bivariate tetramer/dextramer *versus* CD4/CD8 plots. Data were acquired using either a FACSCanto II or a customized FACSAria II (BD Biosciences) and analysed with FlowJo software (TreeStar Inc., Ashland, OR, USA).

### Protein kinase inhibitor treatment

The PKI, dasatinib (Axon Medchem, Reston, VA, USA), was used at a final concentration of 50 nM 19. Briefly, 1 mM dimethylsulphoxide (DMSO) aliquots of PKI were stored at −20°C. Working aliquots of 100 nM were prepared in PBS and, for each sample, an equal volume was added to 25 μl of cells in staining buffer. The cells were then incubated at 37°C in a sealed tube for 30–60 min, after which tetramer or dextramer was added and incubated as described. Of note, we have found that dasatinib stocks are labile when stored at 4°C and therefore recommend storing as one-use frozen aliquots. The effectiveness of the reagent can be tested by confirming an increase in surface expression of TCR and co-receptor on the T cell surface after extended incubations 1.

### Intracellular cytokine staining

Prior to activation, cells were washed from culture medium and incubated for 24 h in ‘resting’ media (RPMI supplemented with 5% FBS, L-glutamine and antibiotics). For activation, cells were incubated at 37°C for 5 h with cognate peptide in 200 μl (3 × 10^6^ cells per ml, 96 round-bottomed culture plates) of resting media containing GolgiStop™ and GolgiPlug™ (both BD Biosciences), according to the manufacturer's instructions. Cells were subsequently stained with a viability dye, equimolar amounts of cognate or irrelevant dextramer and tetramer (6 μg/ml with respect to the pMHC component), and antibodies against cell surface markers (CD8, CD19, CD14 and CD3) as described above for the staining of PBMCs. Cells were prepared for intracellular cytokine staining by incubation with Cytofix/Cytoperm™ (BD Biosciences) according to the manufacturer's instructions (including wash steps), followed by 20 min on ice with mouse αhuman interferon (IFN)-γ antibody (45-15; Miltenyi Biotec). Cells were stored overnight (4°C in the dark) and flow cytometry and data analysis performed as above.

### Fluorescent microscopy

A total of 10^5^ CD8^+^ T cells were labelled with 4′,6-diamidino-2-phenylindole (DAPI) nuclear stain (Life Technologies) and either Alexa488-conjugated peptide-MHC tetramers (at a final concentration of 12·5 μg/ml) or FITC-conjugated dextramers (at a final concentration of 16·25 μg/ml) for 30 min at 37°C or on ice. After three washes with PBS, each sample was fixed in 2% paraformaldehyde. After fixing, the CD8^+^ T cells were resuspended in 100 μl of 2% FBS/PBS and then spun onto microscope slides at 34 × ***g*** for 5 min using a CytoSpin III centrifuge (Harlow Scientific, Arlington, MA, USA). Samples were observed using a Leica DM LB2 (Leica Microsystems, Milton Keynes, UK) fluorescence microscope.

## Results

### Optimized T cell staining with pMHC tetramers and dextramers

We aimed to compare the staining of antigen-specific T cells with pMHC tetramers and dextramers. The dextran backbone of pMHC dextramers means that these reagents can carry a higher number of pMHC and fluorochrome per molecule than pMHC tetramers (Fig. 1a). We first set out to establish the optimal staining conditions for pMHC tetramers and dextramers so that we could compare the optimal detection parameters of each reagent. For this purpose we made use of the ILA1 CD8^+^ T cell clone that recognizes the HLA-A2 restricted ILAKFLHWL epitope from hTERT (residues 540–548). The hTERT epitope is not naturally presented at the tumour cell surface 20, and therefore constitutes a model system that is uncomplicated by the possibility of a natural ligand. Furthermore, we have previously characterized biophysically a wide range of APL for this TCR that range in affinity from K_D_ ∼3 μM to K_D_ ∼2 mM 6,21. This controllable system allows the TCR–pMHC affinity to be varied while other variables such as surface density of TCR and CD8 remain identical. We prepared stocks of HLA-A2–ILAKFLHWL and APL variants thereof, and the human immunodeficiency (HIV)-1 Pol epitope, ILKEPVHGV (residues 476–484), as a non-cognate control. These preparations were used to generate tetramers and dextramers conjugated to the PE fluorochrome. The cognate ILA1 T cell clone was stained with HLA-A2–ILAKFLHWL and HLA-A2–ILKEPVHGV control reagents over a range of pMHC concentrations in order to establish the optimal signal-to-noise ratio for each reagent. A titration of dextramer preparation from 0·037 to 1·2 μg (0·74–24 μg/ml) per test, with respect to the pMHC component, per test, established that 0·3 μg (6 μg/ml) gave the best signal-to-noise ratio (Supporting Information Fig. S1a,b). For tetramer, the optimal signal-to-noise ratio was seen when using 0·5 μg (10 μg/ml) of tetramer per test, with respect to pMHC (Supporting Information Fig. S1c,d). Thus, optimal staining with pMHC tetramers required 1·6-fold more pMHC compared with optimal staining with dextramers. Additionally, the mean fluorescence intensity (MFI) achieved with optimal dextramer staining was more than fourfold greater than could be achieved with tetramer (Supporting Information Fig. S1c). This most probaby reflects the fact that a single dextramer carries multiple fluorochrome molecules. The greater pMHC valency and scaffold flexibility of dextramers compared to tetramers could also increase the cell surface interaction avidity, potentially leading to higher molecular occupancy. Subsequent experiments were performed using the optimal staining conditions for each reagent.

### pMHC-I dextramers exhibit superior fluorescence compared with pMHC-I tetramers

We next set out to compare how pMHC tetramers and dextramers stained T cells over a range of predefined TCR–pMHC affinities. To this end, we again made use of the ILA1 CD8^+^ T cell clone, as this clone has the widest range of biophysically characterized agonist ligands described thus far (K_D_ ∼3 μM to ∼2 mM). Optimized staining of the CD8^+^ ILA1 clone showed that pMHC dextramers gave considerably brighter staining than achieved with pMHC tetramers (Fig. 1b). This difference was most noticeable when the TCR–pMHC interaction was exceptionally weak. The MFI of staining with multimers composed of the 5Y peptide (K_D_ < 250 μM) was more than 75-fold brighter with dextramer than with tetramer (Fig. 1b). pMHC multimers are often used to detect antigen-specific T cells in *ex vivo* samples. In order to mimic this situation in a controlled setting, 10^6^ HLA-A2^+^ PBMC were spiked with 10^4^ ILA1 T cell clone prior to staining (1%). Optimal staining of ILA1 cells with dextramer was substantially brighter in all cases than with the corresponding tetramer (Fig. 1c). This difference became increasingly noticeable when TCR–pMHC interactions were weak, to a point where cells could be sufficiently detected with dextramer of the 5Y variant (K_D_ > 250 μM), but not the corresponding tetramer. Additionally, the admixed ILA1 cells could be detected with dextramer of the 8E variant [K_D_ > 500 μM, and we believe close to 2 mM by extrapolation of response units from surface plasmon resonance (SPR) binding experiments], but not at all with the corresponding tetramer.

### pMHC-II dextramers exhibit improved staining over pMHC-II tetramers

The CD8 co-receptor binds to largely invariant parts of MHC-I and acts to ‘co-receive’ the pMHC-I ligand with the TCR 27,28. The CD4 co-receptor performs a similar role for pMHC-II ligands 29. The CD8 co-receptor binds to MHC-I with an affinity K_D_ ∼100–200 μM 30,31, and acts to enhance the on-rate and slow the off-rate of the TCR–pMHC-I interaction at the cell surface 1,32. The combination of avidities arising from TCR–pMHC-I and pMHC-I–CD8 interactions can substantially impact upon pMHC tetramer staining 6,9,13,15,33. In contrast, pMHC-II–CD4 binding is too weak to be measured by techniques such as SPR 1,32. The current consensus is that the CD4 co-receptor does not play a leading role in stabilizing the TCR–pMHC-II interaction and thus in the binding of pMHC-II tetramers 1,8,34,35. This lack of CD4 ‘co-receptor help’ combined with a weaker TCR interaction ensures that it is considerably more difficult to stain the average CD4^+^ T cell with pMHC multimer than it is to stain the average CD8^+^ T cell 1. Indeed, it is well established that CD4^+^ T cells are notoriously difficult to detect using pMHC tetramer technology 1,8,16.

**Fig. 2:**
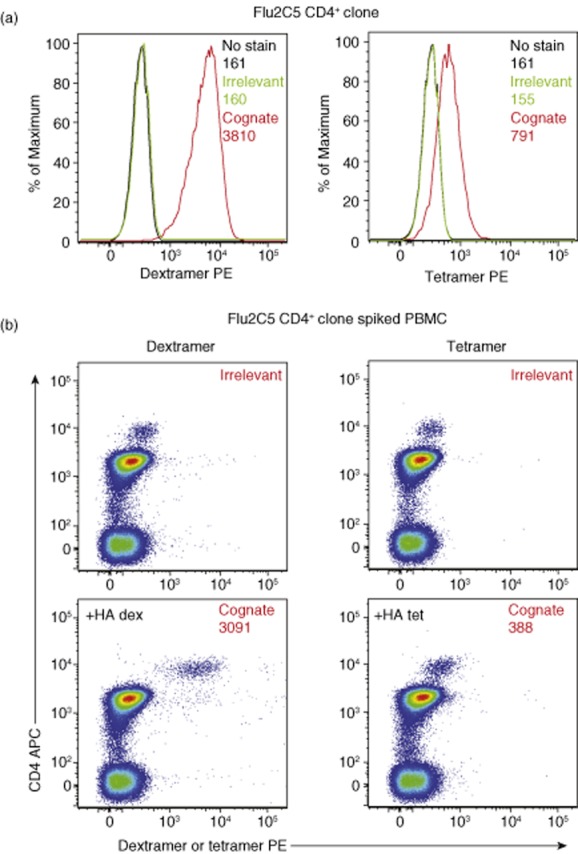
Dextramers made with peptide–major histocompatibility complex (pMHC) class II bind CD4^+^ T cells substantially better than tetramers. (a) The influenza (Flu)2C5 CD4^+^ T cell clone was stained with phycoerythrin (PE)-conjugated (right) or dextramer (left) reagents constructed with human leucocyte antigen (HLA)-DR1 monomers bearing the cognate influenza A virus HA_307–319_ peptide (PKYVKQNTLKLAT; red line) or the HIV-1 p24 Gag_299–312_ peptide (DRFYKTLRAEQASQ; green line) as an irrelevant control. Mean fluorescence intensity (MFI) values are shown inset for each tetramer/dextramer reagent and unstained cells (black line). (b) Human peripheral blood mononuclear cells (PBMCs) (10^6^) were spiked with clonal Flu2C5 cells (10^4^) and stained with the indicated tetramers (right) or dextramers (left), the viability dye Aqua and monoclonal antibodies against the surface markers CD3, CD4, CD8, CD14 and CD19. Gates were set serially on lymphocytes, single cells and live CD3^+^CD14^−^CD19^−^ cells prior to display as CD4 *versus* tetramer/dextramer. MFI values are shown inset.

To determine whether the staining advantage of pMHC-I dextramers over tetramers was also true for the pMHC-II system, we used a CD4^+^ T cell clone (Flu2C5), specific for the HLA-DR1-restricted epitope PKYVKQNTLKLAT (residues 307–319) from HA of influenza virus A. Staining of the Flu2C5 clone with pMHC-II dextramer was more than fivefold brighter than with the corresponding tetramer (Fig. 2a). To examine staining performance in a setting analogous to the *ex vivo* situation, we spiked HLA-DR1^+^ PBMCs with Flu2C5 cells at a frequency of 1%. Of note, the Flu2C5 clone was easily distinguishable in these experiments as a consequence of exceptionally high CD4 expression (Fig. 2b). Again, the cognate pMHC-II dextramer stained Flu2C5 cells readily with sensitivity and specificity, in contrast to the corresponding tetramer (Fig. 2b).

### pMHC dextramers allow high-definition detection of autoimmune T cells

**Fig. 3:**
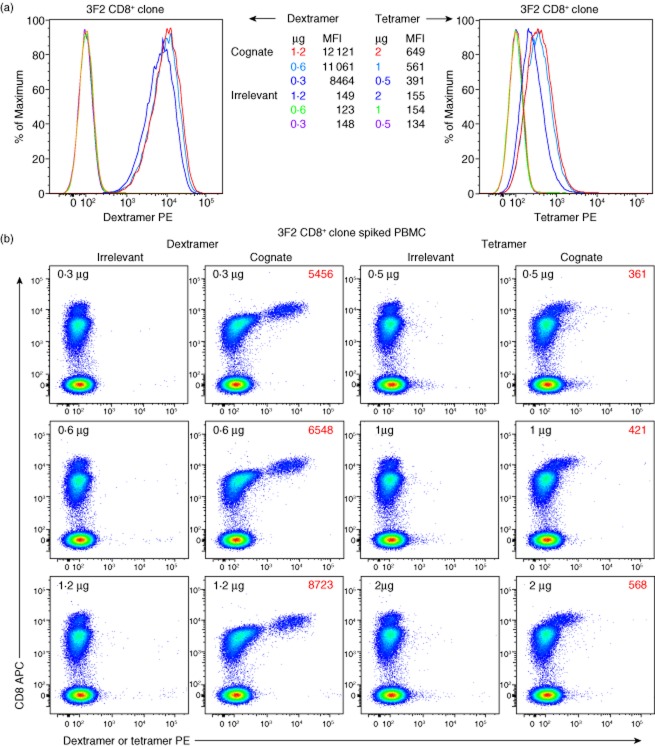
Dextramers bind a type I diabetes-derived CD8^+^ T cell clone substantially better than tetramers and also distinguish such cells from other T cells in a spiked peripheral blood mononuclear cell (PBMC) setting. (a) The 3F2 CD8^+^ T cell clone was stained with phycoerythrin (PE)-conjugated tetramer (right) or dextramer (left) reagents constructed with human leucocyte antigen (HLA)-A2 monomers bearing the cognate preproinsulin (PPI)_15–24_ peptide (ALWGPDPAAA) or the melanoma antigen (MAGE) A3_168–176_ peptide (YLEYRQVPV) as an irrelevant control. Different amounts of tetramer or dextramer were used as indicated with respect to the monomeric peptide-major histocompatibility complex (pMHC) component. Mean fluorescence intensity (MFI) values are shown for each condition. (b) Human PBMCs (10^6^) were spiked with clonal 3F2 cells (10^4^) and stained with the indicated tetramers (right) or dextramers (left), the viability dye Aqua and monoclonal antibodies against the surface markers CD3, CD4, CD8, CD14 and CD19. Gates were set serially on lymphocytes, single cells and live CD3^+^CD14^−^CD19^−^ cells prior to display as CD8 *versus* tetramer/dextramer. Clonal 3F2 cells were easily distinguishable on the basis of brighter CD8 expression. Reagent quantities with respect to the pMHC-I component (black) and MFI values (red) are shown inset.

Autoimmune TCR–pMHC interactions are characterized by poor *in vivo* molecular association between the autoimmune epitope and the MHC or by a weak TCR–pMHC interaction 7,22. Both scenarios can create a serious problem for pMHC multimer staining, as the result of either poor pMHC stability or TCR–pMHC interactions that fall below the threshold for pMHC tetramer staining. In order to examine staining of autoimmune T cells we used a clone specific for the HLA-A2-restricted, PPI-derived epitope, ALWGPDPAAA (residues 15–24). The 3F2 clone was derived from a patient with T1D and is known to bear an identical TCR to the 1E6 and PEAK1 T cell clones grown from the same patient at different time-points 23. This TCR binds to HLA-A2–ALWGPDPAAA with a K_D_ > 250 μM 36. Staining of the 3F2 clone with pMHC dextramer was much greater than 20-fold brighter than could be achieved with pMHC tetramer made with identical pMHC (Fig. 3a). In turn, this enhanced staining allowed clear distinction of these antigen-specific cells when they were spiked into HLA-A2^+^ PBMC at 1% of total cells (Fig. 3b). We thus conclude that pMHC dextramers can be superior to the corresponding tetramers for the detection of autoimmune T cells.

### Dextramers exhibit superior identification of antigen-specific T cells *ex vivo*

**Fig. 4:**
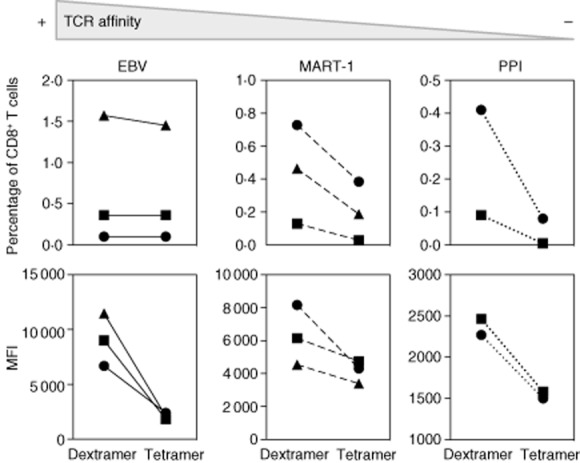
Superior *ex vivo* detection of antigen-specific CD8^+^ T cells with dextramers. Human leucocyte antigen (HLA)-A2^+^ peripheral blood mononuclear cells (PBMCs) from non-type I diabetes (T1D) donors (left, middle) or T1D patients (right) were stained with tetramer or dextramer reagents constructed with HLA-A2 monomers bearing the Epstein–Barr virus (EBV) BMLF-1_259–267_ peptide (GLCTLVAML; left), the MART-1_26–35_ heteroclitic peptide (ELAGIGILTV; middle) or the preproinsulin (PPI)_15–24_ peptide (ALWGPDPAAA; right). Each symbol and line combination denotes a different donor. Gates were set serially on lymphocytes, single cells and live CD3^+^CD14^−^CD19^−^ cells prior to analysis as CD8 *versus* tetramer/dextramer. Detected antigen-specific CD8^+^ T cell frequencies (top) and mean fluorescence intensity (MFI) values (bottom) are shown. Representative flow cytometry plots are shown in Supporting Information Fig. S2. The difference in MFI and percentage of CD8^+^ T cells detected between dextramers and tetramers is significantly different (*P* ≤ 0·01: paired, two-tailed *t*-test).

pMHC multimers are generally used for the detection of T cells in *ex vivo* samples. We next compared how pMHC-I tetramers and dextramers performed in this setting. The affinity of TCR–pMHC interactions tends to exhibit a hierarchy whereby anti-pathogen > anti-self (tumour) > autoimmune 7,37. In order to study each of these categories, we stained HLA-A2^+^ samples from non-T1D and T1D individuals with reagents specific for the epitopes: GLCTLVAML from BMLF-1 (residues 259–267) of Epstein–Barr virus (EBV); E**L**AGIGILTV of MART-1 (residues 26–35: heteroclitic amino acid shown in bold 36); and ALWGPDPAAA of PPI (residues 15–24). HLA-A2-GLCTLVAML dextramer stained an identical proportion of cells to tetramer in two of three samples and slightly more cells in a third sample (1·57% compared to 1·45% of CD8^+^ T cells for dextramer and tetramer, respectively) (Fig. 4). The MFI of staining with dextramers was considerably higher (2·70–5·75 fold increase) in all cases. In contrast, dextramers constructed with HLA-A2-ELAGIGILTV stained almost twice as many cells as the corresponding tetramer (Fig. 4). This difference became even more pronounced when paired HLA-A2-ALWGPDPAAA reagents were used to stain PBMC samples from patients with T1D (Fig. 4 and Supporting Information Fig. S2). Overall, for all the specificities studied in the *ex vivo* setting, the dextramers stained significantly brighter (*P* ≤ 0·01: paired, two-tailed *t*-test) and detected significantly more antigen-specific T cells (*P* ≤ 0·01: paired, two-tailed *t*-test) than tetramers.

### pMHC dextramer staining is enhanced by PKI treatment

**Fig. 5:**
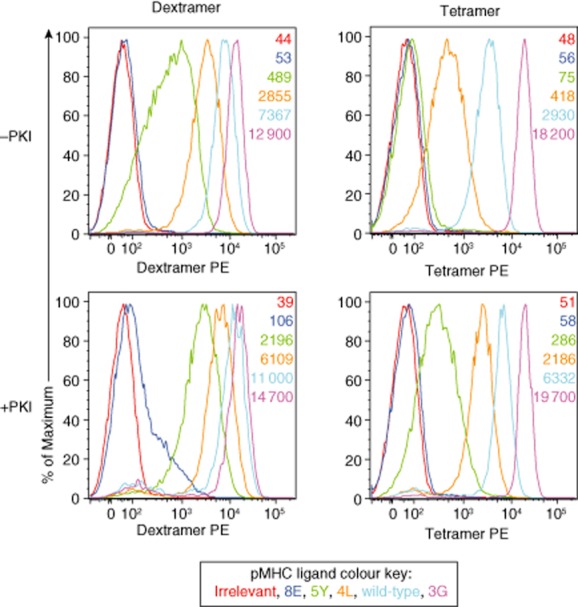
Peptide–major histocompatibility complex (pMHC) dextramer staining is enhanced by protein kinase inhibitor (PKI) treatment. The ILA1 CD8^+^ T cell clone was stained with phycoerythrin (PE)-conjugated tetramer (right) or dextramer (left) reagents constructed with human leucocyte antigen (HLA)-A2^+^ monomers bearing the cognate human telomerase reverse transcriptase (hTERT)_540–548_ peptide (ILAKFLHWL), variants thereof with defined T cell receptor (TCR)-pMHC-I affinities as indicated in the key, or the HIV-1 Pol_476–484_ peptide (ILKEPVHGV) as an irrelevant control after pretreatment with (bottom) or without (top) 50 nM dasatinib. Mean fluorescence intensity (MFI) values are shown inset.

**Fig. 6:**
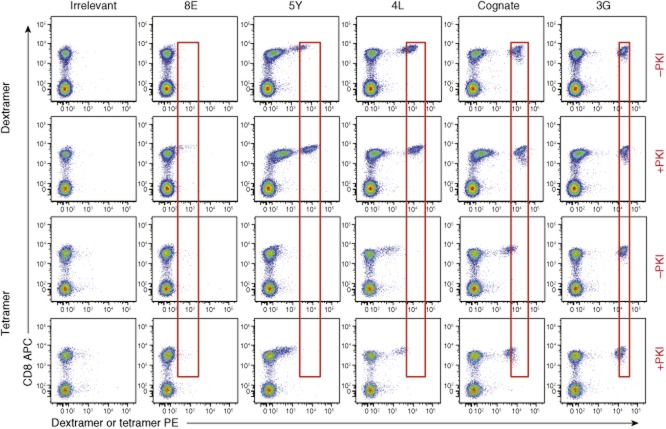
Superior recovery of antigen-specific CD8^+^ T cells with peptide–major histocompatibility complex (pMHC) dextramer + protein kinase inhibitor (PKI). Human peripheral blood mononuclear cells (PBMCs) (10^6^) were spiked with clonal ILA1 cells (10^4^), pretreated with or without 50 nM dasatinib (+/− PKI) and then stained with phycoerythrin (PE)-conjugated tetramer (lower) or dextramer (upper) reagents as indicated (details in Fig. 5), followed by the viability dye Aqua and monoclonal antibodies against the surface markers CD3, CD4, CD8, CD14 and CD19. Gates were set serially on lymphocytes, single cells and live CD3^+^CD14^−^CD19^−^ cells prior to display as CD8 *versus* tetramer/dextramer. In each column, the red box is positioned on the antigen-specific CD8^+^ T cell population that stains most brightly to aid visual comparison.

We have recently described how pMHC staining in the presence of PKI can substantially improve staining with pMHC multimers 1,19. We next sought to determine whether these improvements extended to pMHC dextramers, thereby increasing the range of specific interactions that could be detected. To this end, we stained the ILA1 T cell clone that had been preincubated with or without 50 nM dasatinib for 30 min as indicated (Fig. 5). The MFI of both pMHC tetramer and dextramer staining increased when cells were preincubated with dasatinib in all cases. This difference was most noticeable when the TCR–pMHC affinity decreased, with a maximal effect observed with the 5Y ligand, where PKI treatment increased the MFI by almost 4·5-fold. These results indicate that a combination of pMHC dextramer and 50 nM dasatinib allowed detection of cognate T cells by pMHC multimers with the weakest TCR affinity. Indeed, this combination gave good staining even with the 5Y variant (K_D_ > 250 μM). We next examined the use of pMHC tetramers and dextramers for distinction of T cells within PBMC samples. Figure 6 shows that pMHC dextramer + 50 nM dasatinib enabled better recovery of ILA1 clone spiked into HLA-A2 PBMC when compared to dextramer alone or tetramer ± 50 nM dasatinib. Indeed, for the weak 4L and 5Y ligands (K_D_ = 117 μM and > 250 μM, respectively) a hierarchy of cell recovery emerged of dextramer + PKI > dextramer > tetramer + PKI > tetramer. pMHC tetramer of the 5Y variant alone failed to distinguish an ILA1 population. Conversely, pMHC dextramer of the 8E variant that we believe to have a K_D_ of ∼2 mM was capable of low-level staining in the presence of 50 nM dasatinib.

It is noticeable in Fig. 5 that the dextramer made with the 5Y variant caused some non-specific increase in fluorescence of some CD8^high^ T cells compared to the other reagents or staining with tetramer made with identical pMHC. We have observed this effect with only some pMHC monomers. We do not know the reason for this background, as it only ever occurs with PBMC from a minority of individuals. This effect also occurs in Fig. 3.

**Fig. 7:**
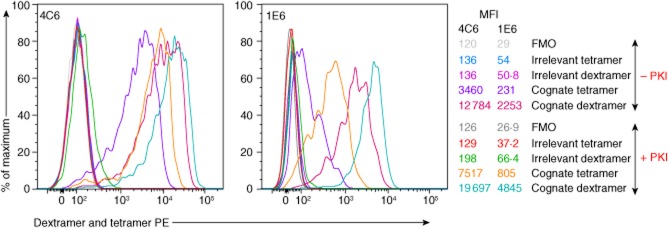
Improved staining of autoimmune T cell clones with peptide–major histocompatibility complex (pMHC) dextramer and protein kinase inhibitor (PKI). The 4C6 and 1E6 CD8^+^ T cell clones were stained with phycoerythrin (PE)-conjugated tetramer or dextramer reagents constructed with human leucocyte antigen (HLA)-A24 monomers bearing the LWMRLLPLL peptide or HLA-A2 monomers bearing the ALWGPDPAAA peptide, respectively, after pretreatment with or without 50 nM dasatinib (+/− PKI). Irrelevant allotype-matched control reagents were constructed with HLA-A24–AYAQKIFKIL and HLA-A2–NLVPMVATV monomers. FMO = fluorescence minus one.

In further experiments, we studied two autoimmune CD8^+^ T cell clones isolated from patients with T1D. These clones kill human pancreatic β cells in a pMHC-I-specific manner 23,24, and bind their respective cognate antigens weakly (4C6, K_D_ = 100 μM; 1E6, K_D_ = 256 μM). In both cases, optimal staining was again observed with the dextramer/dasatinib combination (Fig. 7).

**Fig. 8:**
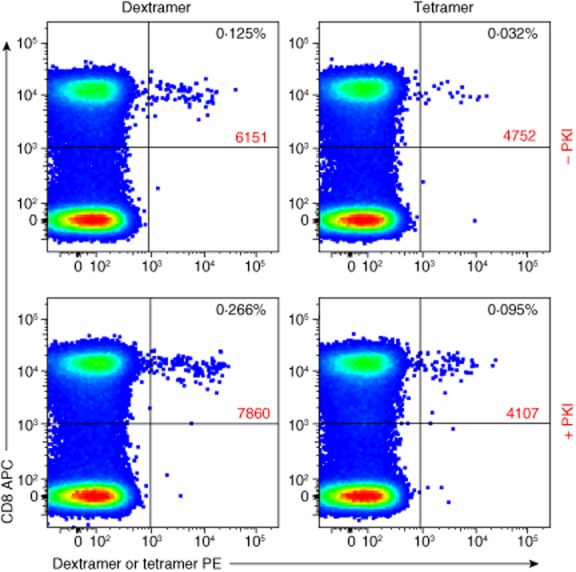
Superior *ex vivo* detection of antigen-specific CD8^+^ T cells with a combinatorial staining approach using a protein kinase inhibitor (PKI) and peptide–major histocompatibility complex (pMHC) dextramer. Human leucocyte antigen (HLA)-A2^+^ peripheral blood mononuclear cells (PBMCs) from healthy controls were stained with phycoerythrin (PE)-conjugated tetramer (right) or dextramer (left) reagents constructed with HLA-A2 monomers bearing the MART-1_26–35_ heteroclitic peptide (ELAGIGILTV) after pretreatment with or without 50 nM dasatinib (+/− PKI). Gates were set serially on lymphocytes, single cells and live CD3^+^CD14^−^CD19^−^ cells prior to display as CD8 *versus* tetramer/dextramer. Detected antigen-specific CD8^+^ T cell frequencies (black) and mean fluorescence intensity (MFI) values (red) are shown inset.

To expand these observations to the *ex vivo* setting, we took advantage of the fact that there can be substantial populations of naive CD8^+^ T cells specific for the HLA-A2-restricted MART-1_26–35_ peptide in the blood of healthy individuals 38. The size of this unique antigen-specific precursor pool appears to reflect epitope recognition by germline-encoded TCR residues 39. In HLA-A2^+^ PBMCs, clear populations CD8^+^ T cells specific for the heteroclitic E**L**AGIGILTV peptide were detected directly *ex vivo* with the cognate pMHC-I tetramer (Fig. 8). Greater numbers of cells were revealed after PKI treatment and the dextramer/dasatinib combination was again optimal, both in terms of population magnitude and staining intensity (Fig. 8).

### pMHC dextramers are compatible with high-resolution flow cytometry

**Fig. 9:**
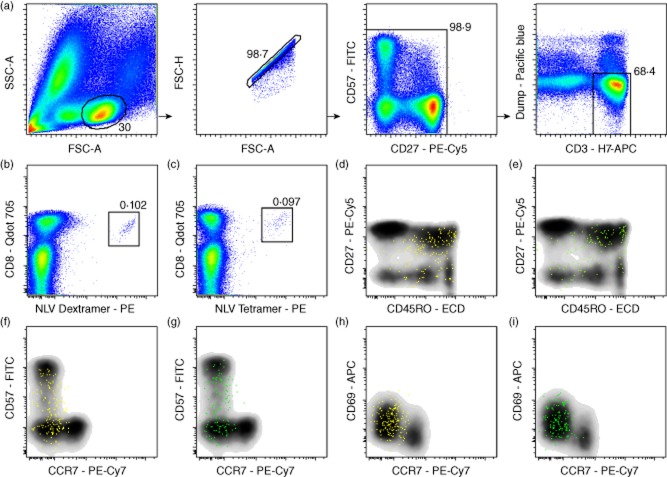
Peptide–major histocompatibility complex (pMHC) dextramers are compatible with high-resolution flow cytometry. Human leucocyte antigen (HLA)-A2^+^ peripheral blood mononuclear cells (PBMCs) from healthy controls were stained with phycoerythrin (PE)-conjugated or dextramer reagents constructed with HLA-A2 monomers bearing the CMV pp65_495–503_ peptide (NLVPMVATV), followed by the viability dye ViViD and monoclonal antibodies against the surface markers CD3, CD8, CD14, CD19, CD27, CD45RO, CD57, CD69 and CCR7. Gates were set serially on lymphocytes, single cells and live CD3^+^CD14^−^CD19^−^ cells (a) prior to display as CD8 *versus* tetramer/dextramer (b,c). Boolean gating was carried out to exclude artefacts and fluorochrome aggregates as indicated (a). Yellow or green dots superimposed on density plots showing the phenotypical profile of the overall CD8^+^ T cell population depict individual antigen-specific CD8^+^ T cells stained with dextramer or tetramer, respectively (d–i). The following bivariate analyses are shown: CD27 *versus* CD45RO (d,e); CD57 *versus* CCR7 (f,g); and CD69 *versus* CCR7 (h,i).

Polychromatic flow cytometry is used frequently in conjunction with pMHC multimers to characterize antigen-specific T cells directly *ex vivo*. As dextramers are substantially bigger than tetramers, it is feasible that they could sterically hinder subsequent staining with fluorescent antibodies. To test this possibility, we stained HLA-A2^+^ PBMCs with reagents specific for the CMV epitope NLVPMVATV (pp65: residues 495–503) followed by a panel of monoclonal antibodies specific for various surface activation and differentiation markers (Fig. 9). The phenotypical data were similar regardless of whether tetramers or dextramers were used to identify the antigen-specific CD8^+^ T cell population. Of note, the enhanced brightness of dextramer-stained cells in the PE channel necessitated minor compensation adjustments (PECy7). Bright compensation controls are therefore advisable for dextramer reagents.

### pMHC dextramers are compatible with intracellular cytokine staining

**Fig. 10:**
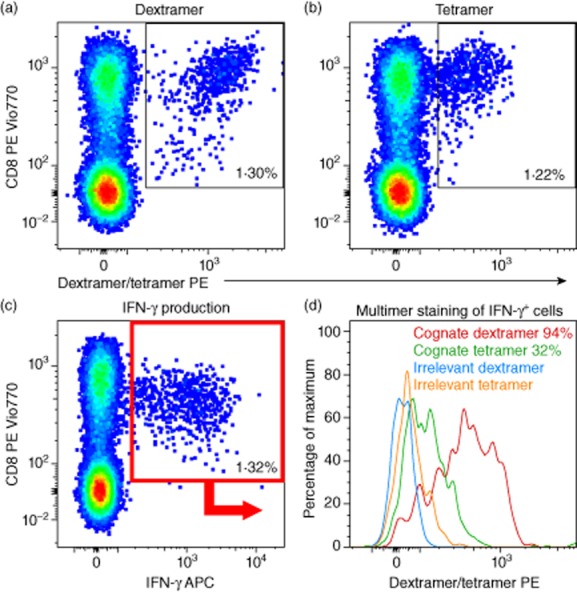
Dextramers can be used to detect activated T cells during an intracellular cytokine assay. A 10-day-old enriched T cell line specific for the influenza epitope, GILGFVFTL (matrix protein residues 58–66), was cultured with or without cognate peptide for 5 h in the presence of monensin and brefeldin A. Cells were subsequently stained with cognate [human leucocyte antigen (HLA)-A2 GILGFVFTL] or irrelevant (HLA-A2–ALWGPDPAAA) dextramer or tetramer, cell surface markers (CD8, CD3, CD19, CD14), a viability stain and intracellularly for interferon (IFN)-γ. Unstimulated cells were stained with cognate dextramer (a) or tetramer (b), with the gate shown based on staining with an irrelevant dextramer or tetramer. The percentage of staining of IFN-γ^+^ cells (c: red gate, based on a fluorescence minus 1) with cognate dextramer (red) or tetramer (green) is based upon the staining of the same IFN-γ^+^ cells with an irrelevant dextramer (blue) or tetramer (orange) (d). The percentage of gated cells shown inset (a–c).

pMHC multimers allow the physical staining of antigen-specific T cells by flow cytometry. Flow cytometry can also be used to separate T cells based on functional activation markers such as CD69 or CD107 that appear transiently on the cell surface after exposure to cognate antigen. Alternatively, antigen-specific T cells can be stained for the accumulation of cytokines in the endoplasmic reticulum after antigenic stimulation (intracellular cytokine staining; ICS). As ICS involves TCR stimulation and concomitant TCR triggering and down-regulation, it results in a lower TCR density at the T cell surface. During the manuscript review process, we were asked whether dextramer staining was compatible with ICS and whether the increased pMHC and fluorochrome payload of pMHC dextramers compared to pMHC tetramers might result in improved staining following ICS. In order to compare the performance of pMHC dextramers and tetramers in conjunction with ICS we used a 10-day-old T cell line primed from a HLA-A2 individual using the influenza epitope, GILGFVFTL (matrix protein residues 58–66). Influenza matrix-specific cells could be detected in this line by flow cytometry using cognate pMHC dextramer and tetramer or by ICS for IFN-γ (Fig. 10a–c). The IFN-γ-positive cells shown in the red gate of Fig. 10c were co-stained with cognate and irrelevant (ALWGPDPAAA: residues 15–24 from PPI) pMHC dextramer and tetramer. Almost all these cells (94%) could be detected with HLA-A2-GILGFVFTL dextramer compared to just 32% recovery with pMHC tetramer made at the same time using the same monomer. We conclude that pMHC dextramers are compatible with ICS and that these reagents are better at recovering antigen-specific cells post-activation than corresponding pMHC tetramers.

### pMHC dextramers are rapidly internalized by cognate T cells

**Fig. 11:**
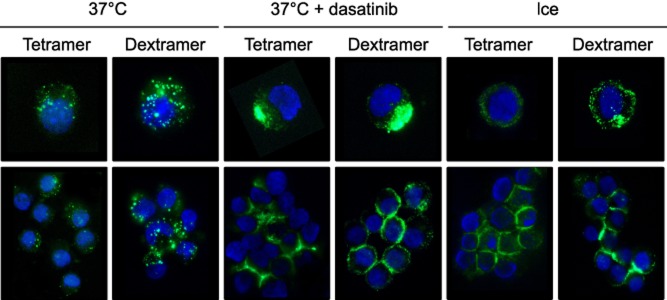
Similar visual staining patterns with peptide–major histocompatibility complex (pMHC) dextramers and tetramers. The NLV2F3 CD8^+^ T cell clone was stained with 4′,6-diamidino-2-phenylindole (DAPI) (blue), followed by Alexa488-conjugated tetramer or fluorescein isothiocyanate (FITC)-conjugated dextramer reagents constructed with human leucocyte antigen (HLA)-A2 monomers bearing the cytomeglovirus (CMV) pp65_495–503_ peptide (NLVPMVATV). Staining conditions are indicated; dasatinib was used at a concentration of 50 nM. Cells were visualized using a Leica DM LB2 fluorescent microscope.

pMHC tetramers are rapidly internalized by antigen-specific T cells 40, ensuring that they can be captured from solution swiftly and irreversibly. We examined staining of NLV2F3 T cells with HLA-A2–NLVPMVATV tetramer and dextramer. Both reagents were rapidly internalized at 37°C (Fig. 11). This internalization was largely inhibited when cells were stained on ice and completely inhibited in the presence of 50 nM dasatinib at 37°C. These experiments confirm that the PKI, dasatinib, prevents internalization of TCRs (and pMHC multimer) during staining and show that tetramer and dextramer staining patterns are very similar despite the greater brightness with dextramer multimers.

## Discussion

In this study we conducted parallel comparative analyses of optimized T cell staining with pMHC tetramers and new dextramer technology assembled with the same pMHC monomer. pMHC tetramers performed well when the monomeric TCR–pMHC affinity was relatively high (K_D_ < 20 μM). TCR interactions of this strength are typical of CD8^+^ T cells that respond to pathogen-derived peptides 7 and pMHC tetramers have excelled for such applications. Our own studies have established that the binding affinity threshold for pMHC-I tetramers is higher than that required for T cell activation 6. As a result, standard pMHC tetramer staining fails to detect all the T cells that are capable of responding to a particular antigen, as T cells with weak, but functional, TCRs might not be detected. This issue becomes especially relevant when using pMHC tetramers to detect T cells that recognize self-derived peptides (tumour-specific and autoimmune T cells) or MHC-II-restricted T cells, as such cells bear TCRs that bind with relatively low affinity 7.

For comprehensive assessment we first made use of the human HLA-A2-restricted T cell clone ILA1, as there is a wide range of characterized analogue ligands for the ILA1 TCR that bind with logarithmically divergent affinities (K_D_ from 3 μM to ∼2 mM). Importantly, all analogues bind to the HLA-A2 molecule with identical affinities 6. The ILA1 T cell clone stained well with both the 3G and wild-type ILA1 ligand (K_D_ 3 μM and 32 μM, respectively), but became increasingly poor with weaker ligands. Parallel staining of ILA1 cells with the corresponding pMHC dextramer gave brighter fluorescence intensity than pMHC tetramer in all cases. This difference became most noticeable with the 4L and 5Y variants that bind with affinities of K_D_ 117 μM and approximately 250 μM, respectively. This difference in staining was manifest when the ILA1 clone was spiked into HLA-A2^+^ PBMC to mimic the conditions of direct *ex vivo* staining allowing a clean detection of the antigen-specific population from the background PBMC with dextramers when TCR–pMHC affinity was low. Given these observations, we conclude that pMHC dextramers perform significantly better than pMHC tetramers for T cell detection when TCR–pMHC affinity is low.

Weak TCR affinities are characteristic of T cells specific for self-derived peptides (tumour-specific and autoimmune T cells) and MHC-II-restricted T cells 7. Given this, we next performed comparisons of pMHC tetramer and pMHC dextramer with MHC-II-restricted T cells and autoimmune T cells in order to test whether dextramers would be better at identifying this subset of antigen-specific T cells. Optimal staining of an HLA-DR1-restricted CD4^+^ T cell clone (Flu2C5) specific for an influenza-derived peptide with pMHC dextramer was more than five times brighter than with the equivalent pMHC tetramer. Increased staining MFI of Flu2C5 with pMHC dextramer compared to tetramer allowed this clone to be clearly distinguishable when it was spiked into HLA-DR1^+^ PBMC. pMHC dextramers stained the autoimmune T cell clone 3F2, which expresses the 1E6 TCR 22,23, with more than 17 times higher intensity than the equivalent pMHC tetramer, allowing clear distinction of these cells from a HLA-A2^+^ PBMC sample. Taken together, these experiments indicate that pMHC dextramers afford considerable advantages over pMHC tetramers for T cell staining. This advantage is particularly distinct when TCR–pMHC affinity is low, allowing detection of MHC-II-restricted and autoimmune T cells.

We next set out to compare pMHC dextramers to tetramers under the conditions that these reagents are often used for the detection of antigen-specific T cells in *ex vivo* samples. We chose to examine staining for T cells specific for viral, tumour and autoimmune antigens in the context of HLA-A2, as the TCRs that bind to such antigens tend to vary in affinity 7. Dextramers and tetramers stained similar numbers of EBV-specific T cells, although staining with dextramers was brighter. Dextramers of a MART-1-derived peptide stained greater numbers of cells than equivalent tetramers. This difference was most exaggerated when pMHC multimers of a PPI-derived peptide were used to stain T cells in the blood of T1D patients. Collectively, these data show that pMHC dextramers outperform pMHC tetramers in identifying antigen-specific T cells *ex vivo*.

We have recently described how the PKI dasatinib can aid staining with pMHC tetramers by preventing down-regulation of the TCR from the T cell surface 19. This inexpensive and easily applied ‘trick’ is especially useful when staining T cells with low-affinity TCRs, such as those that predominate in tumour-specific and autoimmune T cell populations. For competitive assessment of this reagent, four PKI staining conditions were applied to the model ILA1 T cell clone: tetramer ± PKI and dextramer ± PKI. A clear hierarchy of staining intensity was observed, of the order dextramer + PKI > dextramer > tetramer + PKI > tetramer. Staining with dextramer + PKI was 14·6 and 29·4-fold more intense than optimal staining with tetramer with the 4L and 5Y ligands, respectively. We conclude that the highest intensity of staining is seen by using pMHC dextramers in conjunction with PKI. Staining with dextramer + PKI also gave the best distinction of ILA1 cells spiked into HLA-A2^+^ PBMC. Of significant note is that we even observed staining with the 8E variant under these conditions. We estimate that the ILA1 TCR binds to this analogue with a K_D_ of ∼2 mM. Dextramer + PKI also gave the highest staining intensity of HLA-A2 and HLA-A24 CD8^+^ T cell clones isolated from the blood of patients with T1D and specific for preproinsulin-derived peptides. We next took advantage of the fact that there are relatively large populations of naive T cells in HLA-A2^+^ individuals that are specific for the melanoma-derived self-peptide ELAGIGILTV 38. Staining of HLA-A2^+^ PBMC directly *ex vivo* with the four conditions described above showed that the number of cells detected also increased in the order of dextramer + PKI > dextramer > tetramer + PKI > tetramer. We conclude that a combination of dextramer and PKI yields optimal cell identification when targeting T cells that are specific for self-derived peptides.

pMHC tetramers are often utilized in conjunction with fluorochrome-conjugated antibodies for T cell phenotyping. Dextramers are built around a dextran backbone and have considerably larger atomic mass compared with pMHC tetramers. This raised concerns that dextramers might induce steric hindrance against other detection agents when attached to the cell surface and/or alter T cell phenotyping. We tested this concern by phenotyping tetramer- and dextramer-isolated CMV-specific CD8^+^ T cells directly *ex vivo* with a marker for viability and directly conjugated antibodies specific for the surface markers CD3, CD8, CD14, CD19, CD27 CD57, CD45R0, CD27, CD57, CD69 and CCR7 (Fig. 9). Both reagents performed similarly, although once again the dextramer gave much brighter staining than the corresponding tetramer. The enhanced staining observed with pMHC dextramers suggests that it may be possible to utilize these reagents in conjunction with less bright fluorochromes, thereby saving the brightest fluorochromes for the staining of cellular markers with low abundance. Thus, pMHC dextramers might allow cellular phenotyping that would not be routinely possible with pMHC tetramers. Further work will be required in order to formally demonstrate this advantage.

Antigen-specific T cells are often sorted following antigenic stimulation and intracellular cytokine staining. This process involves TCR triggering and down-regulation of TCRs from the cell surface and can result in reduced staining with pMHC tetramers. Direct comparative staining of IFN-γ-positive cells with pMHC dextramers and tetramers following antigenic stimulation of an influenza matrix-specific T cell line showed that pMHC dextramers were able to stain the vast majority of IFN-γ-positive cells. In contrast, fewer than a third of antigen-specific cells by IFN-γ staining could be co-stained with cognate pMHC tetramer. We conclude that recovery of stimulated cells with pMHC dextramer is more efficient than with the corresponding tetramer reagent. This improvement in recovery might be particularly important when using pMHC multimers to confirm the specificity of T cells that activate in ICS assays upon exposure to a specific pathogen, self or cancer target.

Our final aim was to visualize in real time T cells that had been stained under different conditions using both tetramers and dextramers. These studies confirmed our previous findings 40, showing that tetramers are rapidly internalized by antigen-specific T cells at physiological temperature (37°C). The distribution of staining with tetramer and dextramer was similar. Internalization of both tetramer and dextramer was largely inhibited when cells were stained on ice. The pattern of staining with tetramers and dextramers at 37°C + 50 nM dasatinib confirmed that this PKI prevents the internalization of pMHC multimers. Thus, both pMHC tetramers and pMHC dextramers stain cells similarly.

In summary, we rigorously compared pMHC-I and pMHC-II tetramers and dextramers for staining a range of T cells. Both reagents performed well when the TCR affinity was high (e.g. anti-viral CD8^+^ T cells), although the higher valency reagent always stained with higher intensity. This difference in staining intensity became especially noticeable when TCR affinity was low. As we have reported previously, pMHC tetramers struggle to stain T cells where the TCR affinity is lower than K_D_ ∼80 μM 6. Unfortunately, the threshold for T cell activation can be much weaker than this, such that pMHC tetramers sometimes fail to detect T cells capable of responding to the multimerized ligand. This issue is especially problematic when staining T cells that have low-affinity TCRs such as tumour-specific T cells, autoimmune T cells and MHC-II-restricted T cells. Here, we show that the greater pMHC density and fluorochrome load carried by pMHC dextramers circumvents this problem to allow staining of these important T cell populations. Importantly, pMHC dextramers performed well when used for multiplex, polychromatic, T cell phenotyping and in conjunction with ICS. As pMHC dextramers stain T cells much more effectively than the equivalent tetramer, we believe that they could be used with less bright fluorochromes, thereby saving the brightest fluorochomes for staining low-abundance molecules at the T cell surface. In conclusion, our study serves to further highlight that pMHC tetramers fail to stain T cells in cases where the TCR–pMHC interaction is low. As such interactions can still initiate T cell activation 6, this means that pMHC tetramers can fail to identify all T cells that respond to a particular pMHC. The use of pMHC dextramers in conjunction with 50 nM dasatinib allows robust staining of T cells even when the TCR–pMHC affinity is weak (K_D_ > 250 μM), thus enabling ready detection of tumour-specific and autoimmune T cells that might be missed when using pMHC tetramers.

## Supporting Information

**Fig. S1.** Establishing an optimal amount of peptide–major histocompatibility complex (pMHC) dextramer and tetramer to use per test when staining a T cell clone. The ILA1 CD8^+^ T cell clone was stained with different quantities of phycoerythrin (PE)-conjugated tetramer or dextramer reagents constructed with human leucocyte antigen (HLA)-A2 monomers bearing the cognate human telomerase reverse transcriptase (hTERT)_540–548_ peptide (ILAKFLHWL) (a,c) or the HIV-1 Pol_476–484_ peptide (ILKEPVHGV) as an irrelevant control (b,d). Optimal titres with respect to the pMHC-I component (red) were set at the highest signal-to-noise ratio established by comparison of wild-type to irrelevant reagents. Unstained (fluorescence minus one; FMO) controls are shown.

**Fig. S2.** Enhanced *ex vivo* detection of antigen-specific CD8^+^ T cells with dextramers. Dextramers and tetramers were assembled using the same batch of human leucocyte antigen (HLA)-A2 monomer containing epitopes from the BMLF-1_259–267_ protein of Epstein–Barr virus (EBV) (GLCTLVAML) (a), MART-1_26–35_ (E**L**AGIGILTV; heteroclitic amino acid shown in bold) (b) and preproinsulin (PPI)_15–24_ (ALWGPDPAAA) (c). Dextramers and tetramers containing the PPI epitope were used to stain peripheral blood mononuclear cells (PBMC) directly *ex vivo* from HLA-A2^+^ patients with type 1 diabetes (T1Ds) (c), whereas all other PBMC was sourced from HLA-A2^+^ non-TID donors (a,b). The percentage of dextramer or tetramer-positive cells of CD8^+^ T cells and the mean fluorescence intensity (MFI) of this population is displayed in bold. The gates were set on: 1, lymphocytes; 2, single cells; 3, amine-CD3^+^CD14^–^CD19^–^ cells. The subsequent gate was set on CD8 *versus* dextramer and tetramer and events displayed using large dots.

**Fig. S3.** High-resolution version of Fig. 1a.
