# Supplementary material for: Comparison of peptide–major histocompatibility complex tetramers and dextramers for the identification of antigen-specific T cells
Source: Clin Exp Immunol. 2014 Jun 9;177(1):47–63. doi: 10.1111/cei.12339 (PMC4089154; doi:10.1111/cei.12339)
Supplement: Supplementary file 3 — Fig. S3. High-resolution version of Fig. 1a. [file cei0177-0047-SD3.pdf]

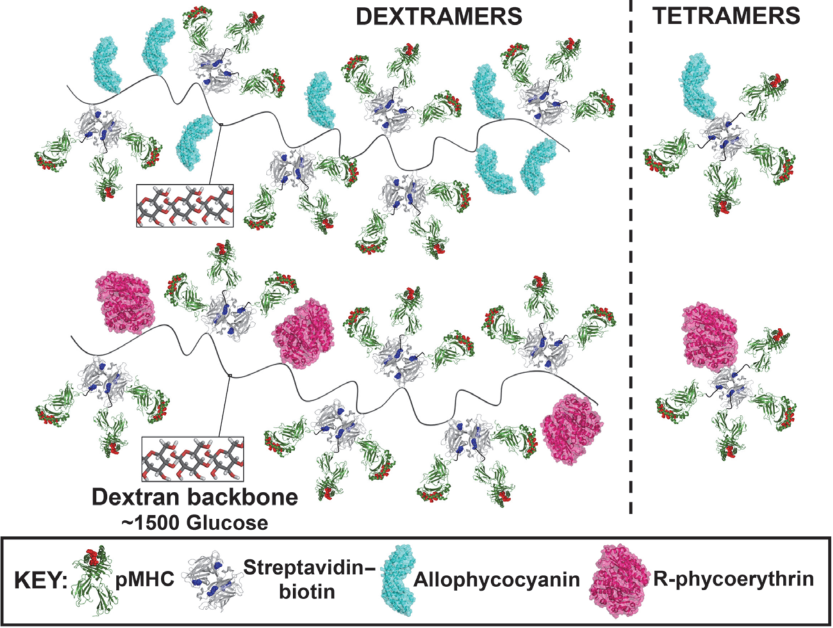


Dolton G, Lissina A, Skowera A, Ladell K, Tungatt K, Jones E, Kronenberg-Versteeg D, Akpovwa H, Pentier JM, Holland CJ, Godkin AJ, Cole DK, Neller MA, Miles JJ, Price DA, Peakman M, Sewell AK. Comparison of peptide–major histocompatibility complex tetramers and dextramers for the identification of antigen-specific T cells. Clin Exp Immunol 2014. doi: 10.1111/cei.12339.
